# Supplementary material for: Defect-evolved quadrupole higher-order topological nanolasers
Source: Nat Commun. 2026 Feb 26;17:3238. doi: 10.1038/s41467-026-70056-4 (PMC13062083; doi:10.1038/s41467-026-70056-4)
Supplement: Supplementary file 1 — Supplementary Information [file 41467_2026_70056_MOESM1_ESM.pdf]

Supplementary Information for

Defect-evolved quadrupole higher-order topological nanolasers

Shengqun Guo[1], Wendi Huang[1], Feng Tian[1], Yufei Zhou[1], Yilan Wang[1], and Taojie Zhou[1,*]

[1] School of Microelectronics, South China University of Technology, Guangzhou, 511442, China

[*] Corresponding author: taojiezhou@scut.edu.cn

Content:

Section S1. Glide symmetry constraints on Wannier bands and nested Wannier bands

Section S2. Fabricated device with flat interface

Section S3. Robustness of the corner state

Section S4. Variation of gap width and field distribution of the corner state

Section S5. Spatial Fourier transformation for the corner and edge states

Section S6. Optical image of the fabricated device

Section S7. Linearly polarized emission characterization

Section S8. Thermal stability for the fabricated devices

Section S9. Lasing at the edge and bulk regions

Section S10. Far-field patterns for corner and edge lasing

Section S11. Time-resolved photo-luminescence measurements

Section S12. Behavior of quadrupole topological nanolaser with various defect parameter

Section S1. Glide symmetry constraints on Wannier bands and nested Wannier bands

The symmetry constraints on Wannier bands and nested Wannier band polarizations can be deduced by analyzing the transformation properties of Wilson loop and nested Wilson loop[1-3]. Under the glide symmetry G_x and G_y, the Wannier band and Wannier band polarizations are constrained as:

$$v_x^j(k_y) \overset{G_x}{=} -v_x^j(k_y)+\frac{1}{2} \mod 1 \tag{S1a}$$

$$v_x^j(k_y) \overset{G_y}{=} v_x^j(-k_y)+\frac{1}{2} \mod 1 \tag{S1b}$$

$$p_y^{v_x^1}(k_x) \overset{G_x}{=} p_y^{v_x^2}(-k_x)+\frac{1}{2},\ p_y^{v_x^3}(k_x) \overset{G_x}{=} p_y^{v_x^4}(-k_x)+\frac{1}{2} \mod 1 \tag{S1c}$$

$$p_y^{v_x^1}(k_x) \overset{G_y}{=} -p_y^{v_x^3}(k_x)+\frac{1}{2},\ p_y^{v_x^2}(k_x) \overset{G_y}{=} -p_y^{v_x^4}(k_x)+\frac{1}{2} \mod 1 \tag{S1d}$$

Hence, the Wannier bands formed in $v_x(k_y)$, $-v_x(k_y)+1/2$, $-v_x(-k_y)$, and $v_x(-k_y)+1/2$. Meanwhile, the presence of the glide symmetry provides a quantization of the composite Wannier bands polarizations constructed by Wannier bands “1+3” and “2+4”, namely, nontrivial polarization of ±1/2 presented in the main text. To further characterize it, we consider a configuration with glide symmetry broken, as shown in Fig. S1. For this configuration, although the Wannier bands are gapped (Fig. S1a), the polarizations of Wannier sectors “1+3” and “2+4” (Fig. S1b) without quantization due to the strong glide symmetry broken perturbations.

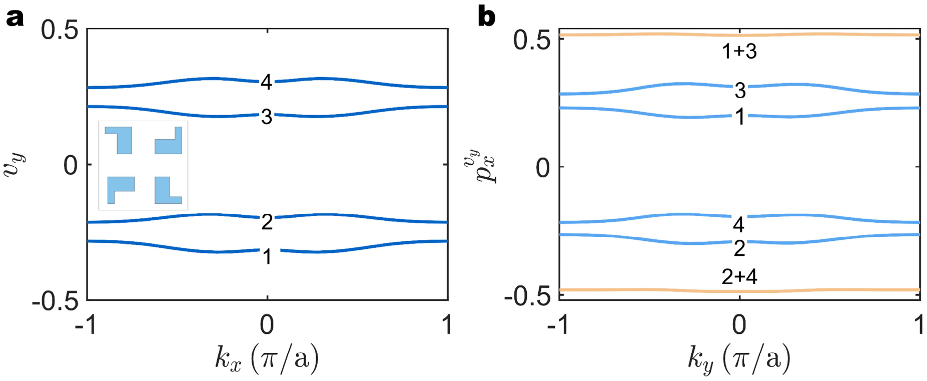

Fig. S1| a Wannier band and **b** the nested Wannier bands for the considered glide symmetry broken configuration.

Section S2. Fabricated device with flat interface

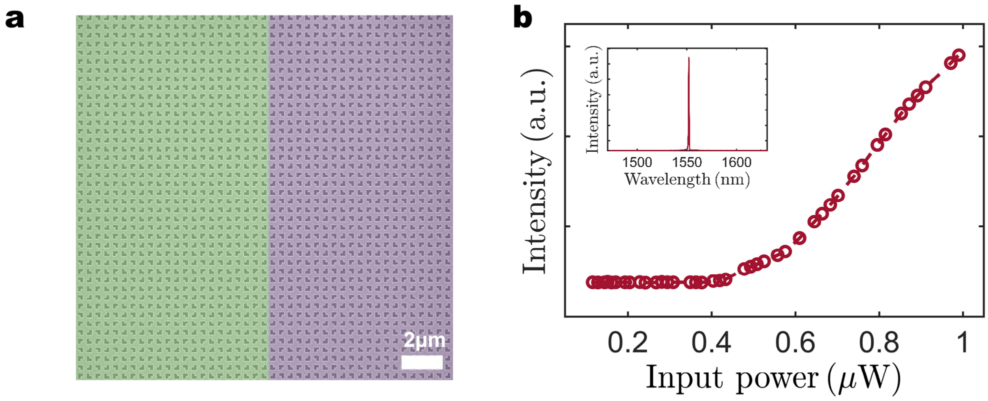

Fig. S2| a SEM image of a fabricated edge state nanolaser with a flat topological interface. **b** *L*-*L* curve and edge state lasing spectra.

Section S3. Robustness of the corner state

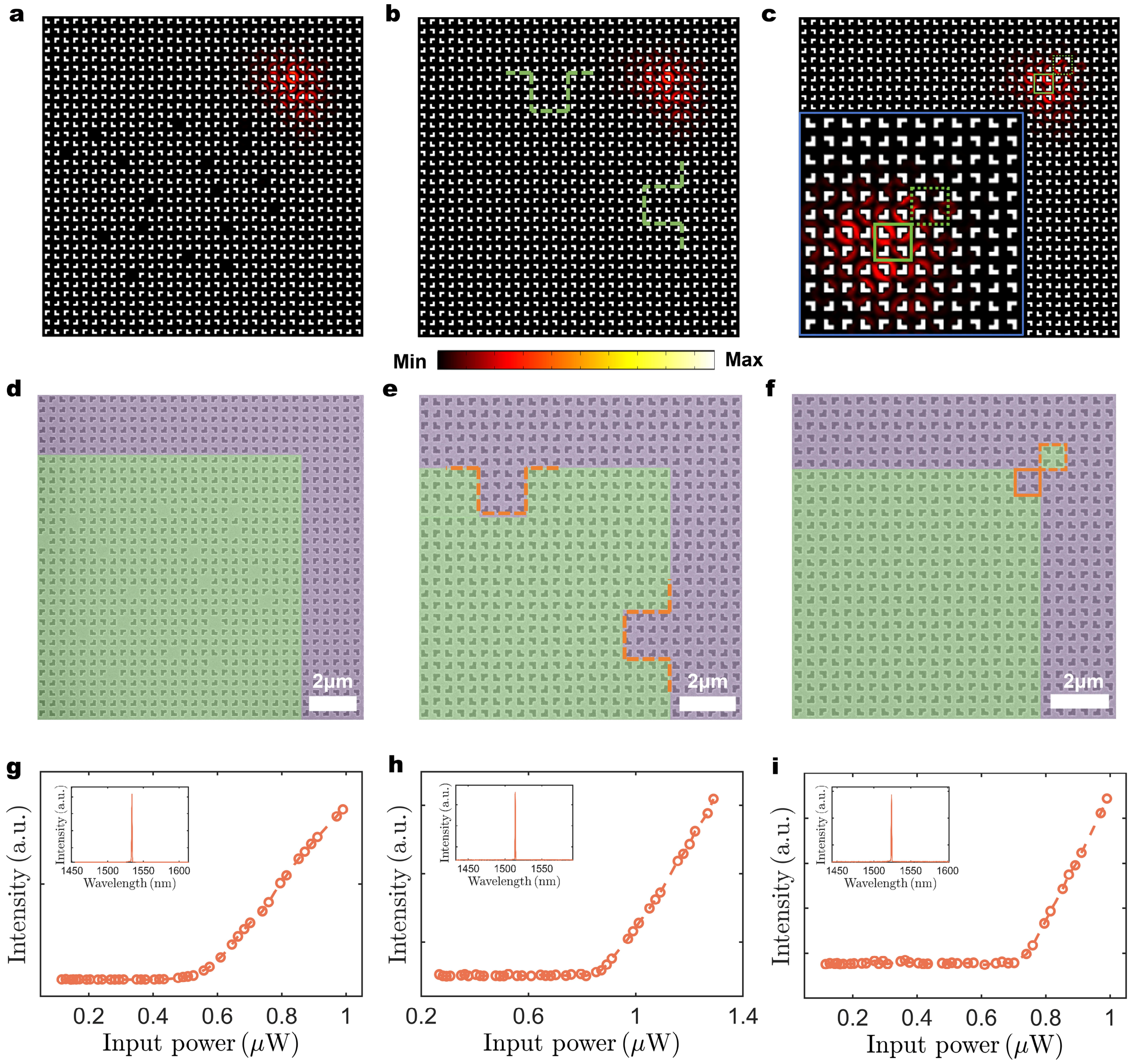

Fig. S3| The robustness of the corner state. Several defect types are considered, including **a** bulk defects, **b** bends, and **c** exchange. **d-f** SEM image of fabricated samples. **g-i** *L-L* curve and lasing spectra for **g** bulk defects, **h** bends, and **i** exchange.

Topological photonics hosts significant advantages in terms of robustness against defects and disorders. In this section, the robustness of the corner state against defects and disorders is conducted. Here, three types of defects are introduced, including eliminating the air holes to generate bulk defects, bends at the boundaries between topologically distinct regions, and exchange of topologically distinct unit cells of the corner region. Figures S3a-c illustrates the corresponding schematics and field distributions of the corner state following the introduction of defects. It is seen that the localized characteristics of the corner state are almost maintained,

still confined to the corner region despite the existence of defects. Figures S3d-f show SEM images of the above three configurations of nanolasers. Despite these introduced defects, lasing from these topological corner state modes is sustained (Figs S3g-i), satisfies the expectations of the previous simulation of robustness.

To estimate the influence of disorder for the corner state, we introduce disorder by adjusting the length s of air holes to $s' = s(1 + \delta_0)$, where δ_0 is a random variable that distributes in the range from $-\delta$ to δ. Figure S4a shows the resonant wavelength response of corner state to the disorder strength δ from 0.01 to 0.1. One can see that the wavelength of the corner state undergoes only slight shifts when the disorder strength changes. For the corner region, we further introduced the disorder strength δ=0.1 (i.e, 10%) both for the position and size of the air holes, where the disorder of each hole is independent, and performed in 10 test configurations, the corner state remains present with a slight wavelength shift, showing the robustness against fabrication disorders.

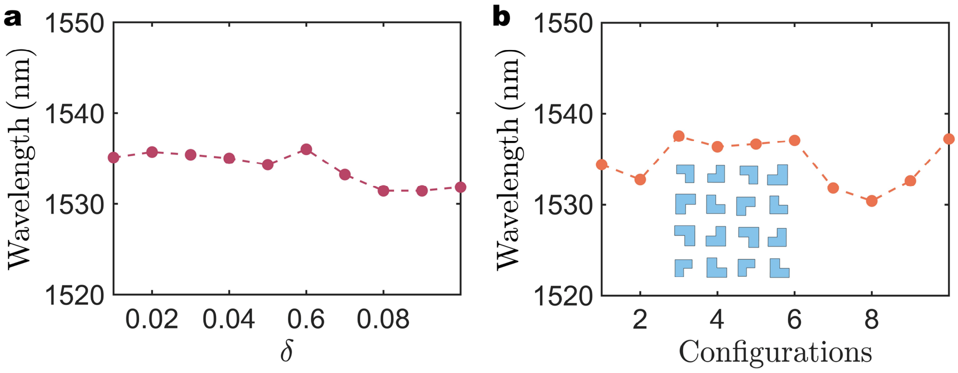

Fig. S4| a Resonant wavelength response to disorder strength δ. The disorder strength δ ranges from 0.01 to 0.1, and other parameters are set to a = 855 nm, s = 0.3a, and Δd_1 = 0.25. **b** Robustness of the corner state by introducing 10% disorder strength into the positions and sizes of air holes in the corner region.

Section S4. Variation of gap width and field distribution of the corner state

Here, we provide more details for the parametric dependence on Δd_1. Figure S5 shows the width of the gap at the Γ point as a function of Δd_1. The gap becomes narrower as the magnitude of Δd_1 decreases, resulting in an increase in the mode volume of the corner state shown in the main text and indicating the phase transition without gap closing at the high symmetry point. The corresponding electric field profiles shown in Fig. S6 exhibit a smoother spatial distribution of corner state as Δd_1 decreases.

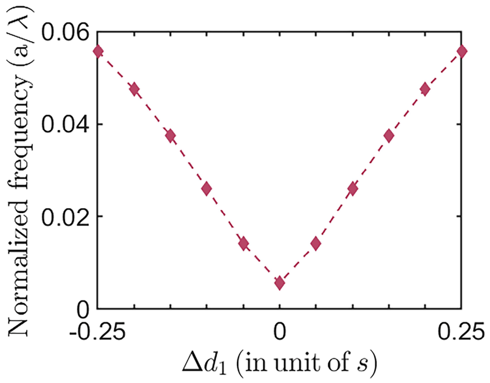

Fig. S5| The frequency difference between the fourth and fifth bands at the Γ point under varying Δd_1.

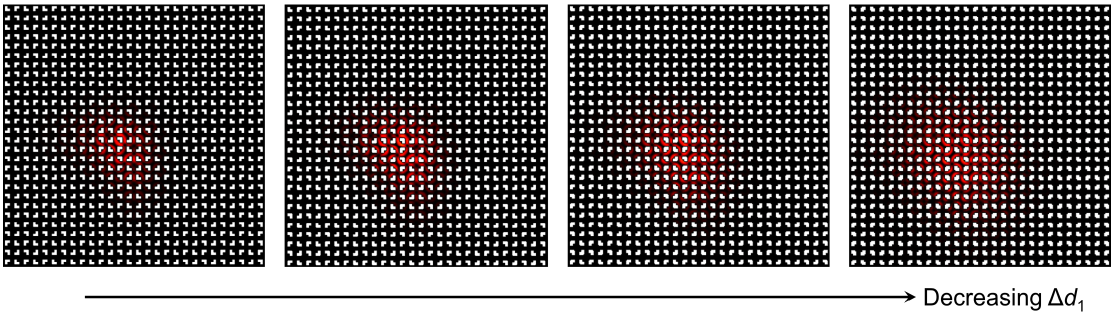

Fig. S6| The simulated electric field profiles of the topological corner state with varying Δd_1. The defect-related parameter Δd_1 ranges from 0.2 to 0.05. The corner state is less localized with decreasing Δd_1.

Section S5. Spatial Fourier transformation for the corner and edge states

The spatial Fourier transforms of the corner state at the corresponding frequency shown in the main text exhibited the negligible wavevector component inside the light cone boundary. Figure S7 present the spatial Fourier transformation of the H_z field for edge states, in which the white circle represents the light cone boundary. It can be observed that the wavevector components of the edge state also mainly distributed outside the light cone, implying the suppressed radiation losses. Figures S8a-b gives the spatial Fourier transformation of the H_z field for corner states under different Δd_1 (0.25 and 0.05) that are amplified to the vicinity of the light cone and normalized, where the light cone boundary is indicated by a white circle. It can be observed that the wavevector components inside the light cone boundary for the cases with $\Delta d_1 = 0.25$ (Fig. S8a) are more prominent compared with $\Delta d_1 = 0.05$ (Fig. S8b), indicating the former hosts more out-of-plane radiation loss, thus resulting in a larger Q-factor for the cases with $\Delta d_1 = 0.05$ despite the expanded mode volume.

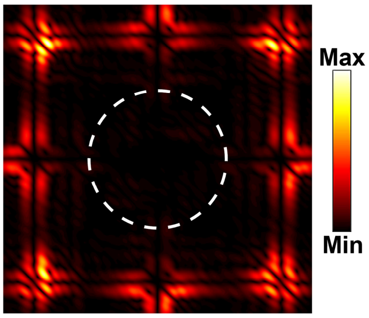

Fig. S7| Spatial Fourier transformation for the edge state.

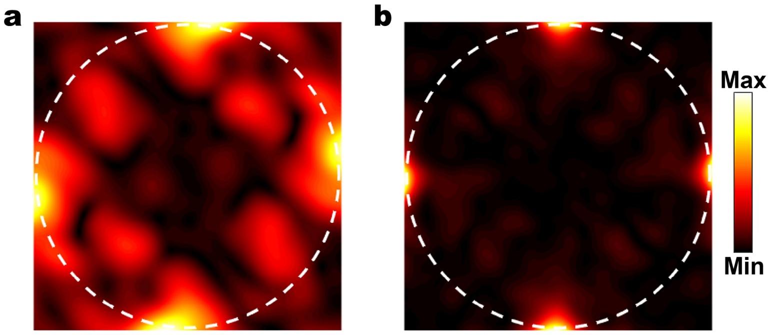

Fig. S8| Spatial Fourier transformation of the H_z field, normalized near the light cone, for corner state at **a** $\Delta d_1 = 0.25$ and **b** $\Delta d_1 = 0.05$.

Section S6. Optical image of the fabricated device

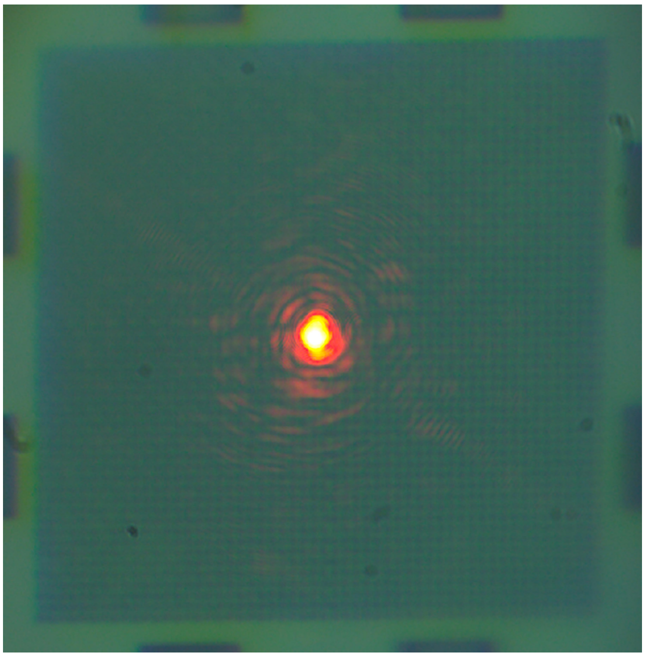

Fig. S9| Topological nanolaser in a home-made μ-PL system. The pumping laser spot is shown in the image, indicating a diameter of around 2 μm and is precisely positioned at the nanocavity by using piezoelectric nanopositioners.

Section S7. Linearly polarized emission characterization

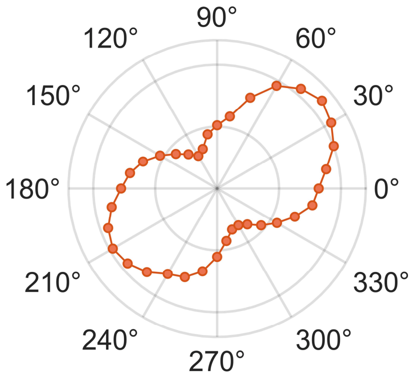

Fig. S10|. Polarization-angle-dependent output intensity for a topological corner state nanolaser.

Section S8. Thermal stability for the fabricated devices

Figure S11a shows the normalized measured lasing spectra under various temperatures from 30 to 70 °C, presenting stable single mode lasing emission as temperature increases. The *L-L* curves of the lasing peak under various temperatures are depicted in Fig. S11b. Figure S11c presents the experimentally determined dependence of lasing threshold on operating temperature. To further estimate the thermal stability, the fabricated device was optically pumped at various pulse widths, while keeping an identical repetition rate of 200 kHz. The normalized lasing spectra for a representative nanolaser under various pulse widths are shown in Fig. S12a, exhibiting stable single mode lasing as pulse width increases from 50 ns to 150 ns. The lasing operation failed when the pulse width was further increased, primarily due to severe thermal issues. Figure S12b shows the *L-L* curves under various pulse width. The significantly increased laser threshold under larger pulse width can be attributed to the reduced gain and increased nonradiative recombination rates. In addition, in experiments, devices from different fabrication batches or from different positions on the wafer may exhibit variations in their laser thresholds.

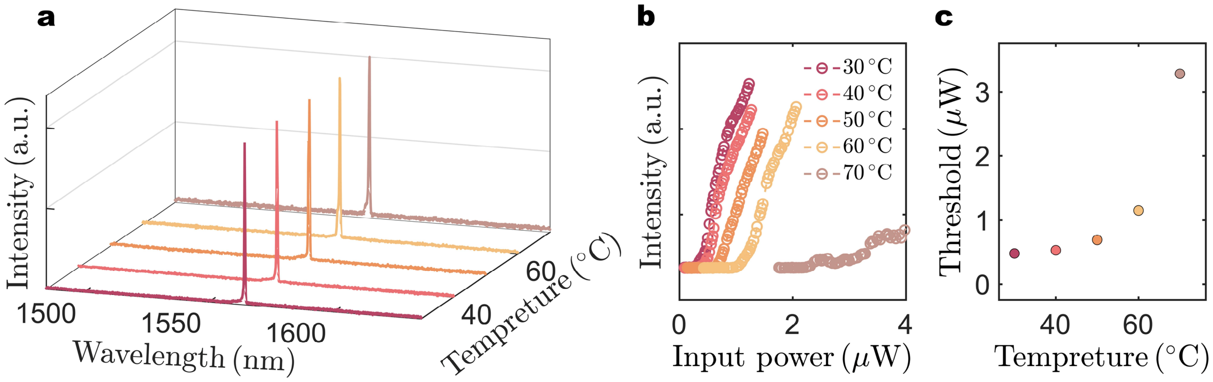

Fig. S11| **a** Temperature-dependent normalized lasing spectra ranging from 30 to 70°C. **b** Temperature-dependent *L-L* curves. **c** Lasing threshold distribution at different temperatures.

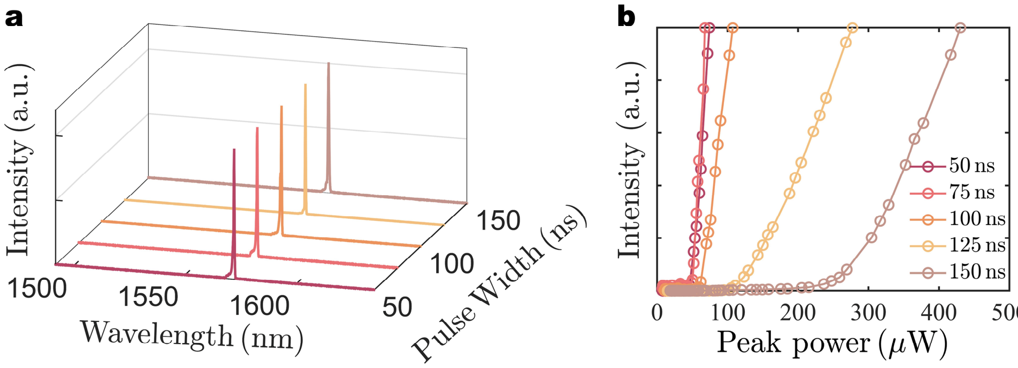

Fig. S12| **a** Pulse width-dependent normalized lasing spectra. **b** Pulse width-dependent normalized *L-L* curves.

Section S9. Lasing at the edge and bulk regions

In addition to the striking corner state, higher-order topological insulators also offer a way for wave trapping in the edge and bulk of single structures. Figures S13a-b display the *L-L* curve for the edge and bulk region of the devices with crossed interfaces, both indicating a clear kink and a low threshold. Moreover, Figs S13c-d depict the near-field optical profiles of edge and bulk lasing below (top panel) and above (bottom panel) lasing thresholds. These results reflect the dimensional hierarchy feature for higher-order photonic topological insulators.

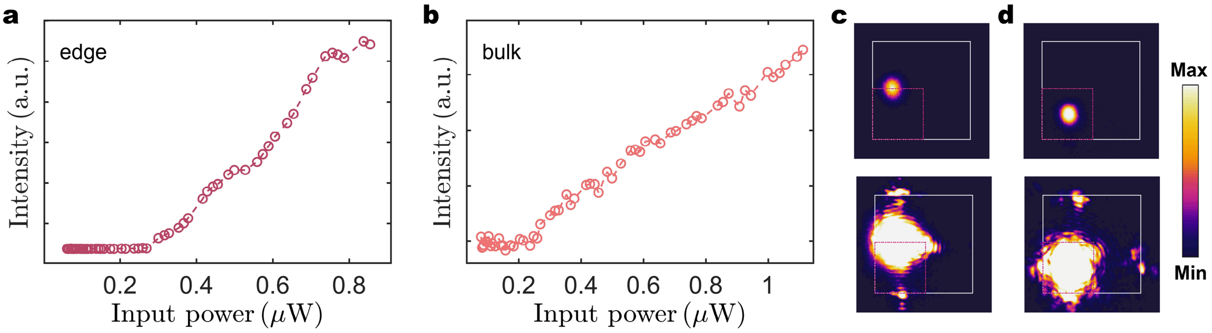

Fig. S13| a-b *L-L* curve of **a** edge and **b** bulk lasing. **c-e** The near-field optical profiles of **c** edge, and **d** bulk lasing measured below and above the lasing threshold.

Section S10. Far-field patterns for corner and edge lasing

a b c

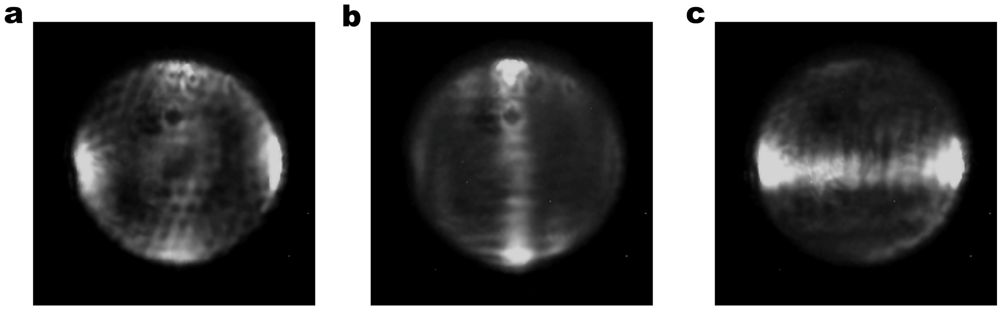

Fig. S14| Far-field patterns for a quadrupole higher-order topological nanolasers (**a**), edge state nanolaser from horizontal interface (**b**), and edge nanolaser from vertical interface (**c**).

Section S11. Time-resolved photo-luminescence measurements

Time-resolved photoluminescence (TRPL) measurements were employed to investigate carrier dynamics. Figure S15a displays normalized TRPL spectra for a representative quadrupole higher-order topological nanolaser, exhibiting a clear transition from spontaneous emission to the coherent stimulated emission with a shorter lifetime(τ_{lasing} =0.26 ns).

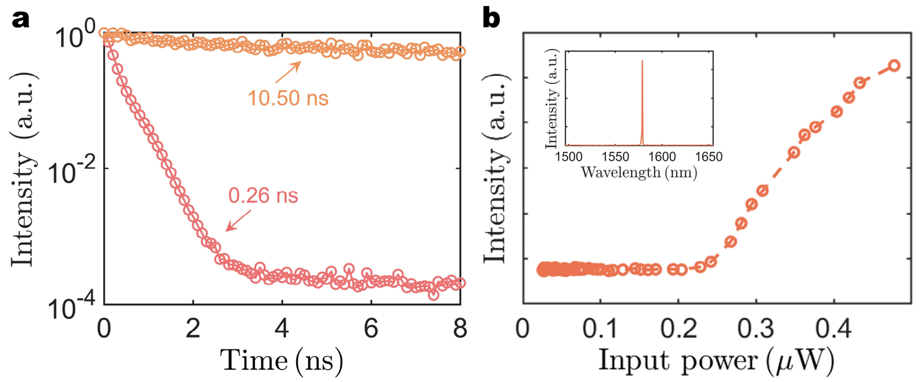

Fig. S15| a Normalized TRPL spectra of the spontaneous emission (orange dots) and stimulated emission (pink dots). **b** *L-L* curve and lasing spectra for the corresponding nanolaser.

Section S12. Behavior of quadrupole topological nanolaser with various defect parameter

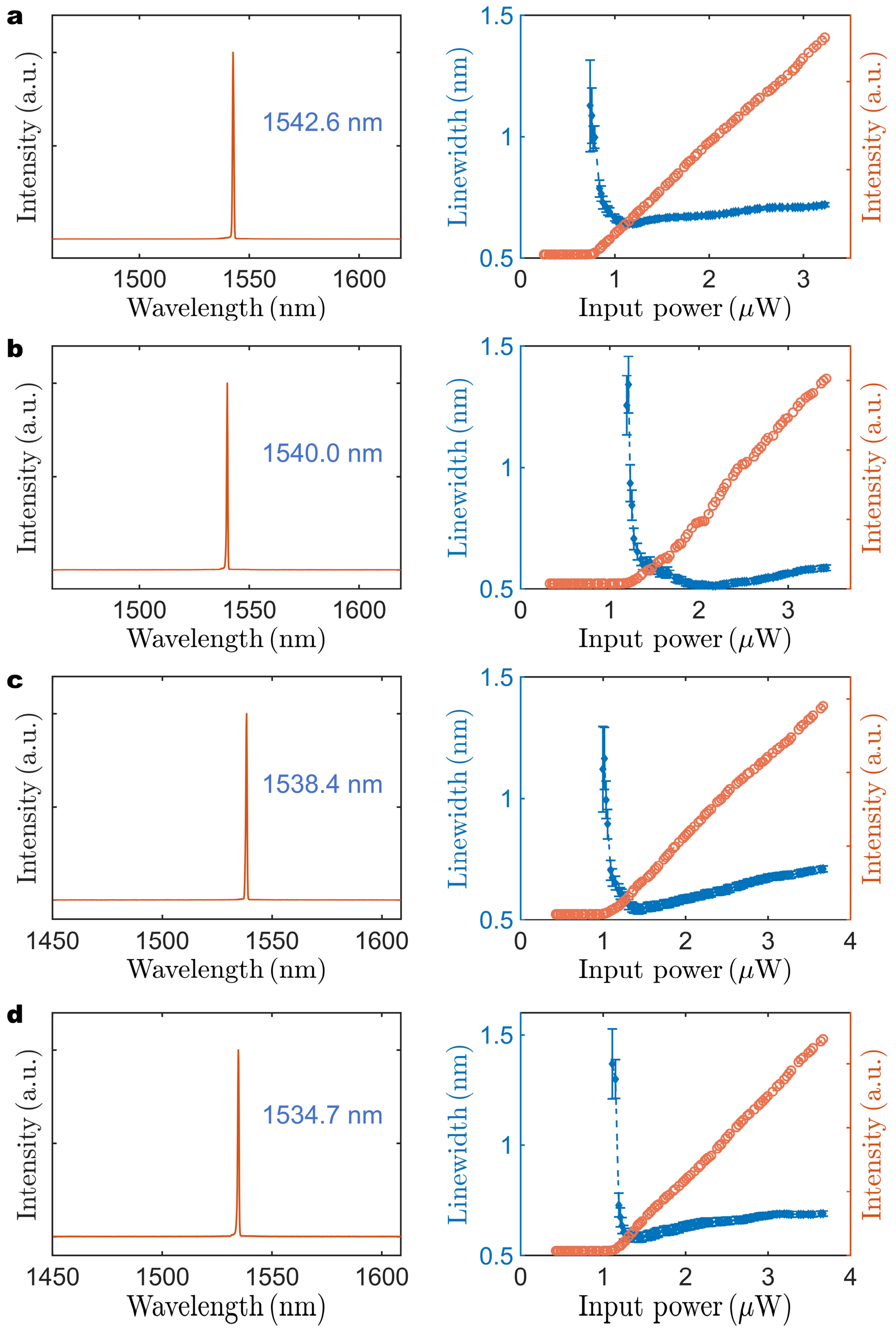

Fig. S16| Lasing spectra, *L*-*L* curve, and linewidth for the proposed nanolasers with defect parameter **a** Δd_1= 0.18, **b** 0.17, **c** 0.16, and **d** 0.15. The error bars indicate the standard errors obtained after fitting.

References

1. W. A. Benalcazar, B. A. Bernevig & T. L. Hughes, Quantized electric multipole insulators. *Science*. **357**, 61(2017).
2. W. A. Benalcazar, B. A. Bernevig & T. L. Hughes, Electric multipole moments, topological multipole moment pumping, and chiral hinge states in crystalline insulators. *Phys. Rev. B*. **96**, 245115(2017).
3. Z.-K. Lin, et al., Anomalous quadrupole topological insulators in two-dimensional nonsymmorphic sonic crystals. *Phys. Rev. B*. **102**, 035105(2020).
