# Supplementary material for: Defect-evolved quadrupole higher-order topological nanolasers
Source: Nat Commun. 2026 Feb 26;17:3238. doi: 10.1038/s41467-026-70056-4 (PMC13062083; doi:10.1038/s41467-026-70056-4)
Supplement: Supplementary file 2 — Reporting Summary [file 41467_2026_70056_MOESM2_ESM.pdf]

nature research

Corresponding author(s): Taojie Zhou

Lasing Reporting Summary

Nature Research wishes to improve the reproducibility of the work that we publish. This form is intended for publication with all accepted papers reporting claims of lasing and provides structure for consistency and transparency in reporting. Some list items might not apply to an individual manuscript, but all fields must be completed for clarity.

For further information on Nature Research policies, including our data availability policy, see Authors & Referees.

ü Experimental design

Please check: are the following details reported in the manuscript?

1. Threshold

Plots of device output power versus pump power over a wide range of values indicating a clear threshold

[x] Yes [ ] No

Fig. 3b, Supplementary Information Figs. S2b, S3g-i, S11b, S12b, S13, S15b, S16.

2. Linewidth narrowing

Plots of spectral power density for the emission at pump powers below, around, and above the lasing threshold, indicating a clear linewidth narrowing at threshold

[x] Yes [ ] No

Fig. 3b, Supplementary Information Fig. S16.

Resolution of the spectrometer used to make spectral measurements

[x] Yes [ ] No

The resolution of spectrometer is ~0.15 nm.

3. Coherent emission

Measurements of the coherence and/or polarization of the emission

[x] Yes [ ] No

Supplementary Information Fig. S10.

4. Beam spatial profile

Image and/or measurement of the spatial shape and profile of the emission, showing a well-defined beam above threshold

[x] Yes [ ] No

Fig. 3c.

5. Operating conditions

Description of the laser and pumping conditions
Continuous-wave, pulsed, temperature of operation

[x] Yes [ ] No

Optical measurement of Method section.

Threshold values provided as density values (e.g. W cm^{-2} or J cm^{-2}) taking into account the area of the device

[x] Yes [ ] No

The threshold is 3.18 kW/cm2 for the demonstrated nanolaser in Fig. 3b taking into account the pumping area.

6. Alternative explanations

Reasoning as to why alternative explanations have been ruled out as responsible for the emission characteristics
e.g. amplified spontaneous, directional scattering; modification of fluorescence spectrum by the cavity

[x] Yes [ ] No

We have systematically studied the lasing properties of our devices, which are shown throughout the manuscript and the Supplementary Information. The L-L curve and linewidth narrowing provide the evidence for lasing emission. Therefore, there are no other explanations as responsible for the emission characteristics.

7. Theoretical analysis

Theoretical analysis that ensures that the experimental values measured are realistic and reasonable
e.g. laser threshold, linewidth, cavity gain-loss, efficiency

[x] Yes [ ] No

Fig. 1, Fig. 2, Fig. 3e, Fig. 4b, Fig. S3.The thresholds and linewidth in main text are obtained from Fig. 3b.

8. Statistics

Number of devices fabricated and tested

[x] Yes [ ] No

Over 20 lasing devices have been fabricated and tested.

Statistical analysis of the device performance and lifetime (time to failure)

[x] Yes [ ] No

The emission properties are stable, in addition to device failure due to time-resolved photo-luminescence measurements and pulse width-dependent lasing behavior measurements.
